# A 5′-Phosphodiester Group Attached to Deoxyguanosine does not Accelerate the Hydrolysis of *cis*-[PtCl(NH_3_)_2_(dGuo)]^3^

**DOI:** 10.1155/MBD.1999.5

**Published:** 1999

**Authors:** Tiphaine Weber, Franck Legendre, Veronika Novozamsky, Jiří Kozelka

**Affiliations:** Laboratoire de Chimie et Biochimie Pharmacologiques et Toxicologiques URA CNRS 400 45, rue des Saints-Pères Paris Cedex 06 75270 France


### A 5'-PHOSPHODIESTER GROUP ATTACHED TO DEOXYGUANOSINE DOES NOT ACCELERATE THE HYDROLYSIS OF *cis*-$[PtCl(NH_3)_2(dGuo)]^+$

Tiphaine Weber, Franck Legendre, Veronika Novozamsky and Jiří Kozelka*

Laboratoire de Chimie et Biochimie Pharmacologiques et Toxicologiques
URA CNRS 400, 45, rue des Saints-Pères, 75270 Paris Cedex 06, France
Fax: +331 42 86 21 75; E-mail: kozelka@citi2.fr

**Abstract**

The influence of the methylphosphoester group on the reversible reaction shown below was studied. Evaluation of the rate constants for the system depicted as well as for the analogous equilibrium involving the nucleoside deoxyguanosine showed that whereas the chloride anation is slowed down by the presence of the methylphosphoester group, the hydrolysis rate constant is not significantly altered. This result speaks against a catalytic role of the 5'-phosphodiester group in the hydrolysis of cisplatin monoadducts with DNA, as suggested previously (Kozelka & Barre, *Chem. Eur. J.* **1997,** *3*, 1405-1409).

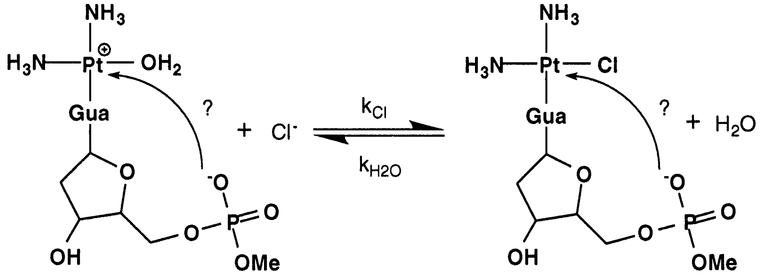

The reaction of Me-5'-dGMP⁻ and dGuo with the diaqua form of cisplatin, *cis*-$[Pt(NH_3)_2(H_2O)_2]^{2+}$, in 0.1 M $NaClO_4$ was also investigated and the corresponding rate constants determined. The phosphodiester group accelerates the replacement of the first $H_2O$ ligand ~10 times, and that of the second $H_2O$ ligand ~2 times.

### 1. Introduction

A number of experimental observations have indicated that the antitumor activity of cisplatin (*cis*-$[PtCl_2(NH_3)_2]$) is related to DNA damage caused by covalent platinum binding to nucleobases [1,2]. The cytotoxic effect is generally ascribed to the major cisplatin-DNA adducts, the 1,2-GG and 1,2-AG intrastrand diadducts, which represent ~85% of all adducts formed upon cisplatin-DNA interaction *in vivo* as well as after DNA platination under certain *in vitro* conditions [3]. The assumption that the 1,2-intrastrand crosslinks are at the origin of antitumor activity is based, on one hand, on the finding that in *E. Coli*, these adducts are indeed cytotoxic [4,5], and on the other hand, on the correlation between the levels of the 1,2-diadducts detected in the white blood cells of cisplatin-treated patients and the patients' response to the treatment, as observed by Reed *et al.* [6]. However, a causal relationship between the 1,2-intrastrand crosslinks and anticancer activity in humans has never been demonstrated.

We have previously hypothesized that the cytotoxic effect of cisplatin could be related to the platinum-DNA monoadducts [7,8]. Our hypothesis was based on the reasoning that the monoadducs, bearing a labile ligand, could "lure" repair proteins and fix them in a covalent DNA-Pt-protein complex. Such a ternary complex formation with the recognition part of the UvrABC excinuclease DNA repair system of *E. Coli* was indeed observed in an experiment by Lambert *et al.* [9]. Another indication that the monoadducts could be related to antitumor activity was the finding that asymmetrical *cis*-diaminedichloro complexes with bulky substituents, forming with DNA long-lived monoadducts, showed enhanced cytotoxicity [7,9]. Recently, Natile's and Farrell's groups reported substantial *in vitro* cytotoxicity and *in vivo* antitumor activity of *trans*-$[PtCl_2L_2]$ complexes, with L being E-imino-ether [10,11] or quinoline [12,13]; these complexes are characterized by relatively long-lived monoadducs with DNA [14-16].

There is conclusive evidence showing that *cis*-$[PtCl_2(NH_3)_2]$ does not react with DNA directly but through a solvent-assisted pathway [17-19]. Less clear is whether the monoaquated cisplatin form, *cis*-$[PtCl(NH_3)_2(H_2O)]^+$ (**1**), or the diaqua complex, *cis*-$[Pt(NH_3)_2(H_2O)_2]^{2+}$ (**2**), is the major species undergoing the DNA platination reaction. Whereas DNA monoadducts formed upon the reaction with **2**, bearing an aqua ligand, can rearrange to diadducts (chelates) directly, the chloro-monoadducts resulting from a reaction between DNA and **1** have to be hydrolyzed to aqua-monoadducts before further ligand substitution by a nucleobase [19-21]. Thus, the chloro-monoadducts have considerably longer lifetimes than the aqua-monoadducts [19,22-25]. The rate of hydrolysis of the former has been shown to depend on the adjacent bases, in particular on the base 5' to the platinated guanine [26]. We have recently suggested that this sequence-dependence could be due to a catalytic action of the phosphodiester group flanking the monoadduct from the 5' side. Either a nucleophilic catalysis [27] or a general base catalysis are conceivable mechanisms; in both cases, the exact positioning of the phosphodiester group, which depends on the nature of the adjacent bases, would be expected to determine the hydrolysis rate.

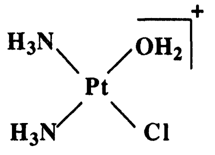

**1**

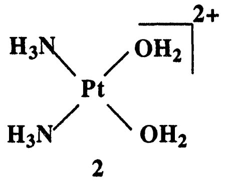

**2**

In order to test the possible involvement of the 5'-phosphodiester group in the hydrolysis mechanism, we have studied in this work the reversible chloride anation of the mononucleotide complex *cis*-[Pt(NH$_3$)$_2$(H$_2$O)(Me-5'-dGMP)]$^+$ (**3**) [equation (1)]. The complexes **3** and **4** are models for respectively aqua- and chloro-monoadducts formed between cisplatin and DNA. The hydrolysis and chloride anation rate constants, $k_{H2O}$ and $k_{Cl}$, were compared to those determined in a parallel study of the analogous reaction (2),

$$\underset{\mathbf{3}}{cis\text{-}[Pt(NH_3)_2(H_2O)(Me\text{-}5'\text{-}dGMP)]^+} + Cl^- \underset{k_{H2O}}{\overset{k_{Cl}}{\rightleftharpoons}} \underset{\mathbf{4}}{cis\text{-}[PtCl(NH_3)_2(Me\text{-}5'\text{-}dGMP)]} + H_2O \quad (1)$$

$$\underset{\mathbf{5}}{cis\text{-}[Pt(NH_3)_2(H_2O)(dGuo)]^{2+}} + Cl^- \underset{k_{H2O}}{\overset{k_{Cl}}{\rightleftharpoons}} \underset{\mathbf{6}}{cis\text{-}[PtCl(NH_3)_2(dGuo)]^+} + H_2O \quad (2)$$

where the nucleotide Me-5'-dGMP was replaced by the nucleoside deoxyguanosine. We were thus able to quantify the influence of the phosphodiester group on $k_{H2O}$ and $k_{Cl}$.

In addition, we have followed the formation of **3** from **2** and Me-5'-dGMP, and that of **5** from **2** and deoxyguanosine, and determined the appropriate rate constants. The reaction between Me-5'-dGMP and **2** and the reversible anation of **3** (Eq.1) were followed using $^1$H NMR, whereas the analogous reactions with dGuo were analyzed by means of reverse-phase HPLC. In the NMR-monitored runs, pH changes during the reactions were accounted for mathematically, while in the reactions followed by HPLC, the pH was kept constant. Advantages and disadvantages of both methods are discussed.

### 2. Experimental

*cis*-[Pt(ND$_3$)$_2$(D$_2$O)$_2$]$^{2+}$ (**2**) was prepared by dissolving *cis*-[Pt(NO$_3$)$_2$(NH$_3$)$_2$] [28,29] in D$_2$O. 2'-deoxyguanosine (dGuo) was purchased from Sigma.

2'-deoxyguanosine-5'-monophosphate-methylester (Me-5'-dGMP) was prepared as ammonium salt by an adaptation of the method of Miller *et al.* [30]. 760 mg Dicyclohexylcarbodiimide (DCC, Aldrich) were added to a suspension of 5'-dGMP (free acid, Sigma, 250 mg) in 100 mL of methanol (Carlo Erba, pro analysis) and the mixture was stirred 48 h at room temperature. The solvent was evaporated under vacuum to ~3 mL. DCC and its by-products were precipitated by addition of 50 mL of water and filtered off in two steps: first, using filter paper, and second, passing through a D4 glass frit (filtering over frit directly congests the frit). The filtrate was extracted with 3x20 mL of cyclohexane, evaporated under vacuum to ~10 mL and the rest lyophilised to dryness. The colorless microcrystalline material obtained was dissolved in ~3 mL D$_2$O and the solution passed through a column filled with ~0.5 mL of a Dowex®-50 resin (Sigma) charged with NH$_4^+$ and rinsed with D$_2$O. The purity of the collected fractions was checked using $^1$H NMR. The fractions with satisfactory purity were assembled and lyophilized. The final product was stored under argon at -32 °C. A thermoanalysis revealed a 5.73% weight loss in the temperature range between 30 and 120 °C, with a maximum rate at 70 °C, and a 15.13% weight loss between 120 and 250 °C, with a maximum rate at 170 °C. The first step was completely reversible when the sample was kept under ambient atmosphere, and was attributed to 1.5 equivs. of "adsorbed" H$_2$O (theor.: 6.54%). During the second, irreversible step, the sample turned black, indicating decomposition. Anal. (deuterated sample): Calcd. for $C_{11}H_{11}D_8N_6O_7P \cdot 1.5\ D_2O$: C, 31.74; N, 20.19%. Found: C 31.10; N, 19.48%.

The samples for the kinetic runs to be followed by NMR were prepared by weighing all quantities (including the liquid components) using a semimicro balance (precision ±0.01 mg). All reactions were carried out in 0.1 M $NaClO_4$ at 20 °C. The $^1H$ NMR spectra were recorded on a Bruker ARX 250 spectrometer with 3-trimethylsilyl(2,2,4,4-$D_4$)propionate as reference. The HDO peak was suppressed by means of presaturation.

The reaction between **2** and deoxyguanosine was followed using the HPLC-based method described by Gonnet *et al.* [31]. The samples withdrawn at different time intervals were quenched by addition of KCl in excess and by cooling down to liquid nitrogen temperature. The pH was kept within 4.5±0.1 by addition of $HClO_4$. The HPLC analysis was performed using a Beckman 126 pump coupled to a Beckman diode array detector 168 and a System Gold V810 integrator. The system was connected to a Rheodyn 7125 valve. A cation exchange HPLC column Nucleosil SA, 250 x 4.6 mm, ID 5 mm (Colochrom, France) was employed, the mobile phase was sodium $NaClO_4$ 0.25 M (pH 4.4 adjusted by $HClO_4$) for 15 minutes and a gradient to 0.5 M for 30 minutes, flow rate 1 mL/min; column temperature 50°C. The detection wavelength of 258 nm was that of the quasi-isosbestic point of the overall reaction. The elution times increased with increasing positive charge of the eluted species, i.e., deoxyguanosine < *cis*-$[PtCl(NH_3)_2(dGuo)]^+$ (**6**) < *cis*-$[Pt(NH_3)_2(dGuo)_2]^{2+}$.

For the kinetic analysis of the reversible hydrolysis of **6**, the same reaction was carried out with **2** in twofold excess over deoxyguanosine, so that the yield of **6** was maximized. After one hour reaction time at room temperature, the same volume of saturated NaCl solution was added to the reaction mixture in order to replace all aqua ligands with chloride. The mixture was subjected to cation exchange HPLC separation using the same conditions as described above, and **6** collected at the outlet was immediately cooled to liquid nitrogen temperature. This solution, which was 0.5 M in $NaClO_4$, was subsequently used to follow the establishment of the hydrolysis equilibrium (Eq. 2). The reaction was started by diluting 5 times with water, warming up to 20 °C and adjusting the pH to 4.5±0.1 by addition of $HClO_4$. The pH was kept within this range by eventual addition of NaOH. The total content in deoxyguanosine was quantified spectrophotometrically by measuring the absorbance at the quasi-isosbestic point (258 nm), with the molar absorption coefficient $\varepsilon_{258}$ determined as 12300 $M^{-1}cm^{-1}$ from a deoxyguanosine solution of a known concentration. The kinetics of the reversible hydrolysis of **6** to **5** was followed by withdrawing aliquots and analyzing them immediately using the same cation exchange HPLC system. Six independent experiments were carried out. An exactly determined quantity of NaCl (~1 equiv. with respect to **6**) was added to the solution of **6** at the beginning of two experiments.

### 3. Results

### 3.1. Kinetics of the reaction between 2 and Me-5'-dGMP

The reaction scheme is depicted in Scheme 1. The guanine H8 resonances of the species **3** ($8.30 \le \delta_{H8} \le 8.57$; see Figure 1) and **7** ($\delta_{H8}$ = 8.42 ppm) are downfield from that due to the free ligand ($\delta_{H8}$ = 8.08 ppm); integration of the H8 peaks could therefore be used for concentration measurements. The chemical shift of H8(**3**), which is sensitive to the deprotonation of the aqua ligand ($pK_{a3}$ = 7.03±0.06 in $D_2O$) as well as to that of the guanine N1 atom ($pK_a$(N1) = 9.16±0.06 in $D_2O$), has been utilized as an internal pD indicator (Figure 1)[§]. We have followed the reactions between **2** and Me-5'-dGMP (**L**) in two different runs: i) in acidic milieu, where the reaction along $k_{L1}$ was preponderant, and ii) in neutral medium, where a major fraction of **2** was deprotonated to **2-D** and thus the pathway involving $k_{L2}$ was more important.

The establishment of the protolytic equilibria is, of course, rapid, therefore, the integration of NMR peaks allows only the sum $[\mathbf{3_{tot}}] = [\mathbf{3}] + [\mathbf{3\text{-}D}]$ to be determined. The differential equations for the time-derivatives of [**L**], [$\mathbf{3_{tot}}$], and [**7**] are shown below along with that for [$\mathbf{2_{tot}}$], defined as $[\mathbf{2_{tot}}] = [\mathbf{2}] + [\mathbf{2\text{-}D}] + [\mathbf{2\text{-}2D}]$:

$$d[\mathbf{L}]/dt = -(k_{L1}[\mathbf{2}] + k_{L2}[\mathbf{2\text{-}D}] + k_{BIS}[\mathbf{3}])[\mathbf{L}] \quad (3a)$$

$$d[\mathbf{2_{tot}}]/dt = -(k_{L1}[\mathbf{2}] + k_{L2}[\mathbf{2\text{-}D}])[\mathbf{L}] \quad (3b)$$

$$d[\mathbf{3_{tot}}]/dt = (k_{L1}[\mathbf{2}] + k_{L2}[\mathbf{2\text{-}D}] - k_{BIS}[\mathbf{3}])[\mathbf{L}] \quad (3c)$$

$$d[\mathbf{7}]/dt = k_{BIS}[\mathbf{3}][\mathbf{L}] \quad (3d)$$

Numerical integration of these differential equations yields the theoretical concentration curves.

Obviously, the calculation of the time-derivatives (Eq. 3a-d) requires the concentrations of the specific protolytic forms, [**2**], [**2-D**], and [**3**], to be defined. This can be achieved using two different approaches, one applicable in the acidic solution, and the other in the neutral milieu, as explained in the following paragraphs.

§ Conversion of these $pK_a$ values obtained in $D_2O$ solution to $H_2O$ according to Martin [32] yields $pK_{a3}$ = 6.46 and $pK_a(N1)$ = 8.58.

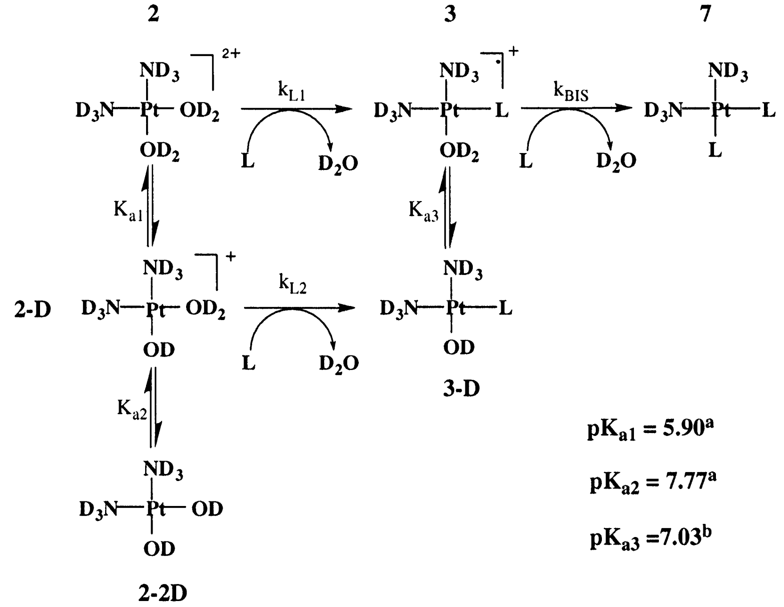

**Scheme 1.** Reaction between *cis*-$[Pt(ND_3)_2(D_2O)_2]^{2+}$ (**2**) and Me-5'-dGMP (**L**) in $D_2O$.

[a] extrapolated for $D_2O$ from [32] and [33].

[b] determined from the titration curve in Figure 1.

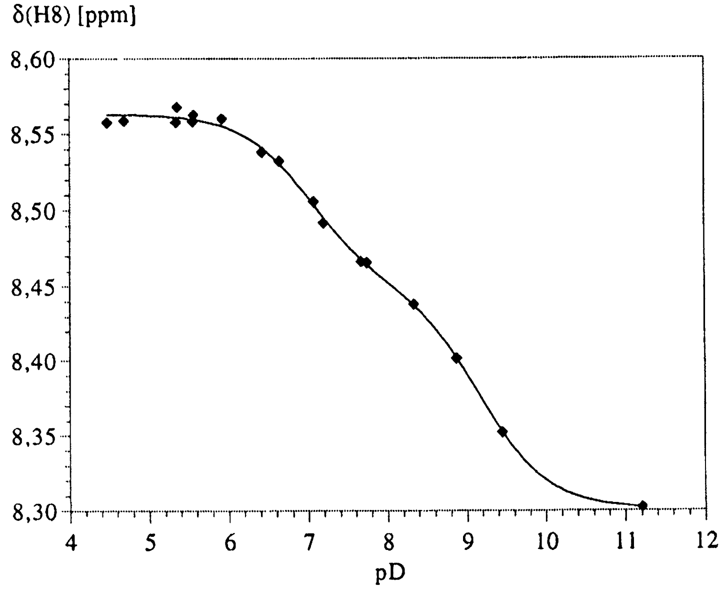

**Figure 1.** The H8 chemical shift of *cis*-$[Pt(ND_3)_2(D_2O)(Me\text{-}5'\text{-}dGMP)]^+$ (**3**) in $D_2O$ as a function of pD. The full line represents a double-sigmoidal function $\delta(H8)\,[ppm] = p + q[10^{(s-pD)}/(1 + 10^{(s-pD)})] + r[10^{(t-pD)}/(1 + 10^{(t-pD)})]$, with p = 8.301 ppm, q = 0.152 ppm, r = 0.110 ppm, s = 9.158, t = 7.028. p, p+q, and p+q+r represent the H8 chemical shifts of the unprotonated, monoprotonated, and doubly protonated forms of **3**, respectively; s and t are the $pK_a$ values corresponding to the (de)protonation of the guanine N1 and of the aqua ligand, respectively.

### *3.1.1. Reaction in acidic medium*

When approximately stoichiometric, exactly determined quantities of **2** and **L** were mixed in an NMR tube so that the initial concentrations were ~4 mM, the pD of the reaction mixture remained within the limits 5.3≤pD≤5.6 in the course of the reaction. In these conditions, the protolytic equilibrium $K_{a2}$ (Scheme 1) could be neglected, and dissociation of **2** ($K_{a1}$) and of **3** ($K_{a3}$) could be considered as the only sources of $D^+$. The reduced system of equations characterized by the rate constants $k_{L1}$, $k_{L2}$, $k_{BIS}$, and the equilibrium constants $K_{a1}$ and $K_{a3}$ leads to a cubic equation for [**2-D**]:

$$a[\mathbf{2\text{-}D}]^3 + b[\mathbf{2\text{-}D}]^2 + c[\mathbf{2\text{-}D}] + d = 0 \quad (4)$$

with

$$a = K_{a3} - K_{a1}$$

$$b = K_{a3}[\mathbf{3_{tot}}] + K_{a1}(K_{a3} + [\mathbf{2_{tot}}] - K_{a1})$$

$$c = K_{a1}[\mathbf{2_{tot}}]\,(2K_{a1} - K_{a3})$$

$$d = K_{a1}^2[\mathbf{2_{tot}}]^2$$

and

$$[\mathbf{2_{tot}}] = [\mathbf{2}] + [\mathbf{2\text{-}D}] \qquad [\mathbf{3_{tot}}] = [\mathbf{3}] + [\mathbf{3\text{-}D}] \qquad [\mathbf{2\text{-}2D}] = 0$$

From the three theoretical roots of Eq.(4) [34], the physically meaningful one was found to be that shown in Eq.(5):

$$[\mathbf{2\text{-}D}] = -2Q^{1/2}\cos[(\theta+4\pi)/3] - b/3a \quad (5)$$

with

$$Q = [(b/a)^2 - 3c/a]/9$$

$$\theta = \arccos(R/Q^{3/2})$$

$$R = [2(b/a)^3 - 9bc/a^2 + 27d/a]/54$$

From the balance for $[D^+]$, we have

$$[D^+] = K_{a1}([\mathbf{2_{tot}}] - [\mathbf{2\text{-}D}])/[\mathbf{2\text{-}D}]$$

$$[\mathbf{2}] = [\mathbf{2\text{-}D}][D^+]/K_{a1}$$

$$[\mathbf{3\text{-}D}] = [D^+] - [\mathbf{2\text{-}D}]$$

$$[\mathbf{3}] = [\mathbf{3_{tot}}] - [\mathbf{3\text{-}D}]$$

For any given set of instantaneous concentrations [**L**], [$\mathbf{2_{tot}}$], [$\mathbf{3_{tot}}$], and [**7**], we can thus express their time-derivatives (Eq. 3a-d) by means of the rate constants $k_{L1}$, $k_{L2}$, $k_{BIS}$, the equilibrium constants $K_{a1}$ and $K_{a3}$, and the concentrations [**L**], [$\mathbf{2_{tot}}$], [$\mathbf{3_{tot}}$], and [**7**] themselves.

*3.1.2. Reaction in neutral milieu*

In a second experiment, approximately stoichiometric, exactly determined quantities of **2** and **L** were mixed in an NMR tube with an equimolar amount of NaOD, so that the initial concentrations were ~8 mM. The initial pD was 6.7 and increased to 7.7 in the course of the reaction. In this case, neither equilibrium of Scheme 1 could be neglected, and a purely analytical determination of $[D^+]$, [**2**], [**2-D**], and [**3**] became impossible. We then took advantage of the fact that the pD range lie in the buffer zone of **3** ($pK_{a3}$ = 7.03 in $D_2O$), and could thus be monitored using the chemical shift of H8(**3**) (Figure 1) with precision. A fit function of the form

$$[D^+] = p + q\exp(-r\,t^{1/2}) \quad (6)$$

(t is the time in seconds), with the coefficients (optimized using the program Kaleidagraph) p = $2.2968 \times 10^{-8}$ M, q = 1.7583 M, and r = 0.014189 $s^{-1/2}$, was found to describe very well the experimental $[D^+]$-time curve (Figure 2). The fit function (6) enabled us to convert the experimental $[D^+]$-time curve into an analytical expression for $[D^+]$, from which the remaining concentrations required for the differential equations (3a-d) could easily be determined:

$$[\mathbf{2\text{-}D}] = [\mathbf{2_{tot}}]/([D^+]/K_{a1} + K_{a2}/[D^+] + 1)$$

$$[\mathbf{2}] = [\mathbf{2\text{-}D}][D^+]/K_{a1}$$

$$[\mathbf{3}] = [D^+][\mathbf{3_{tot}}]/(K_{a3} + [D^+])$$

*3.1.3. Evaluation of the platination rate constants*

While in acidic medium, **L** reacts almost exclusively with the diaqua form **2** (pathway along $k_{L1}$), in neutral medium, **2** converts preponderantly to the aqua-hydroxo form **2-D**, and the reactions of the latter (along $k_{L2}$) become important. In order to obtain precise and consistent values for $k_{L1}$, $k_{L2}$, and $k_{BIS}$, the experimental curves for [**L**] and [$\mathbf{3_{tot}}$] were fitted simultaneously for both reactions (Figure 3). (Since [**7**] is related to [**L**] and [$\mathbf{3_{tot}}$] *via* the constant sum of all the H8 peak integrals, fitting its curve would be redundant.) The optimized rate constants are given in Table 1 together with those determined for the platination of **2** with deoxyguanosine (Section 3.3).

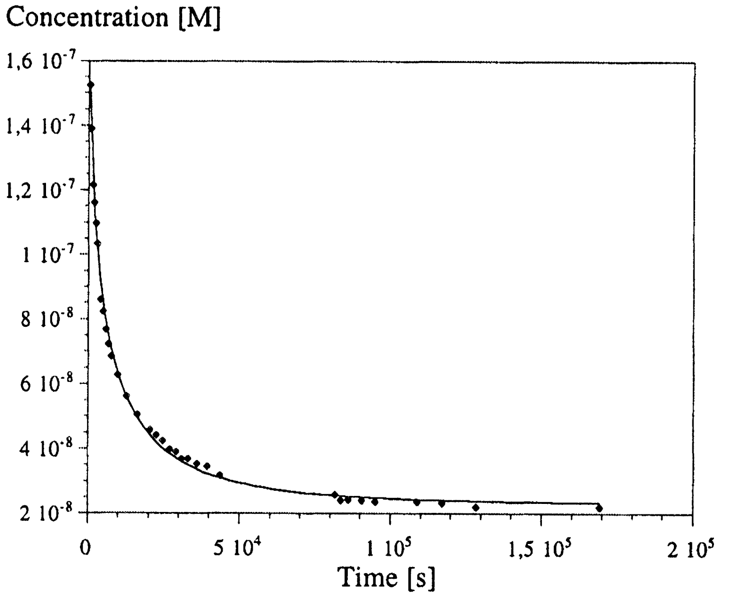

**Figure 2.** Concentration of $D^+$ measured for the reaction run between Me-5'-dGMP⁻ and **2** in neutral solution, as a function of time (♦). The H8 chemical shift of **3** was used as pD indicator (see Figure 1). The solid line represents the fit function $[D^+] = p + q\ \exp(-r\ t^{1/2})$, with the optimized coefficients p = 2.2968x10$^{-8}$ M, q = 1.7583 M, and r = 0.014189 s$^{-1/2}$.

3.2. Kinetics of the reversible chloride anation of **3**

In this part of the work, we have followed the establishment of the equilibrium between **3** and **4** (Eq.1) in 0.1 M $NaClO_4$ at 20 °C from approx. stoichiometric amounts of **3** and NaCl. For this purpose, **2** was first allowed to react with 1 equiv. of Me-5'-dGMP (Section 3.1.1). At the end of the reaction, the exact

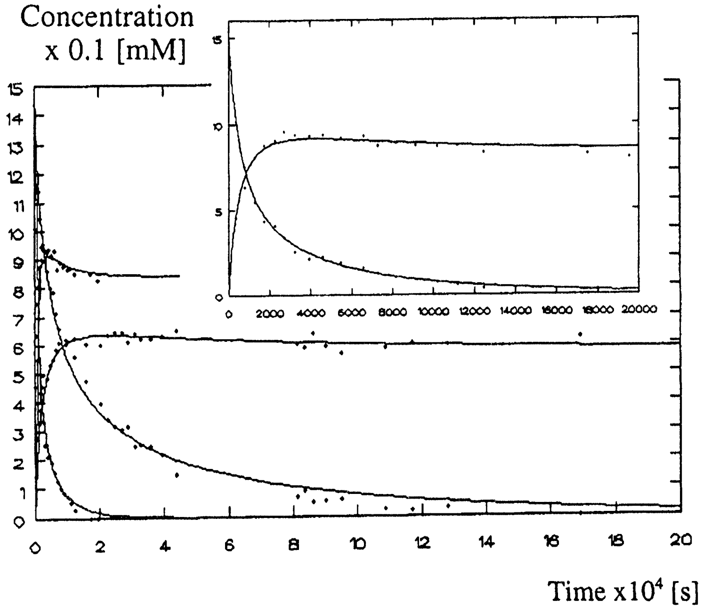

**Figure 3.** Experimental (+) and calculated (full line) concentration curves for Me-5'-dGMP⁻ (**L**) (descending curves) and $3_{tot}$ (ascending curves), for the reactions between **L** and **2** in acidic (faster reaction, terminated after ca. 2x10$^4$s, shown enlarged in the inset) and neutral (slower reaction, followed until ca. 17x10$^4$s) aqueous 0.1 M $NaClO_4$ at 20 °C. The rate constants $k_{L1}$, $k_{L2}$, and $k_{BIS}$ were iterated in order to fit simultaneously all four curves.

**Table 1.** Rate constants for the platination of *cis*-$[Pt(NH_3)_2(H_2O)_2]^{2+}$ (**2**) with deoxyguanosine and Me-5'-dGMP. Standard deviations are given in parentheses.

| | dGuo | Me-5'-dGMP$^-$ |
|---|---|---|
| $k_{L1}$ $[M^{-1}s^{-1}]$ | 0.117(1) | 1.18(3) |
| $k_{L2}$ $[M^{-1}s^{-1}]$ | n.d. | 0.016(3) |
| $k_{BIS}$ $[M^{-1}s^{-1}]$ | 0.073(2) | 0.129(3) |

n.d., not determined concentrations of **3**, **7**, and of the remaining starting complex **2** were evaluated, and a precisely determined amount of NaCl (approx. 1 equiv. with respect to **3**) in a small volume of $D_2O$ was injected to the NMR tube. The guanine H8 signals of **3** and **4** were again used for concentration measurements. The H8 chemical shift of **3** was constant (8.576±0.004 ppm) during this experiment, indicating that the pD of the reaction mixture remained inferior to 5. The H8 chemical shift of **4** (8.45 ppm) was well separated from that of **3**, but overlapped with that of **7** (8.42 ppm). However, since the concentration of **7** did not change during the chloride anation of **3**, the integral under the peak of **7** could be evaluated at the beginning of the reaction and then subtracted from the sum **4**+**7**.

The kinetic study of the reversible chloride anation of **3** *in situ* was complicated by the presence of the unreacted diaqua complex **2** remaining in the tube. This complex can undergo chloride anation to *cis*-$[PtCl(ND_3)_2(D_2O)]^+$ and, in a second step, to *cis*-$[PtCl_2(ND_3)_2]$, and acts therefore as scavenger of $Cl^-$. It was therefore necessary to take the two-step anation of **2** (Eq.7) explicitly into account.

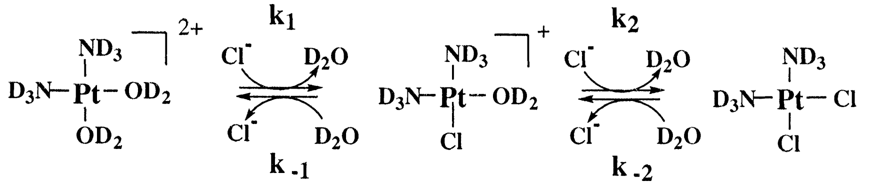

(7)

**2** **8** **9**

Since the mixture remained acidic (pD ≤ 5) during the reaction, the deprotonation of **2-D** ($K_{a2}$) and of **3** ($K_{a3}$) (Scheme 1) as well as that of **8** ($pK_a$ = 6.96; extrapolated for $D_2O$ from [33]) could be neglected. The only protolytic equilibrium that has been taken into account was thus that between **2** and **2-D** ($K_{a1}$, Scheme 1).

The differential equations to be integrated are thus according to the equations (1) and (7):

$$d[\mathbf{3}]/dt = -k_{Cl}[\mathbf{3}][Cl^-] + k_{H2O}[\mathbf{4}] \quad (8a)$$

$$d[Cl^-]/dt = -k_{Cl}[\mathbf{3}][Cl^-] + k_{H2O}[\mathbf{4}] - k_1[\mathbf{2}][Cl^-] + k_{-1}[\mathbf{8}] - k_2[\mathbf{8}][Cl^-] + k_{-2}[\mathbf{9}] \quad (8b)$$

$$d[\mathbf{2}_{tot}]/dt = -k_1[\mathbf{2}][Cl^-] + k_{-1}[\mathbf{8}] \quad (8c)$$

$$d[\mathbf{8}]/dt = k_1[\mathbf{2}][Cl^-] - k_{-1}[\mathbf{8}] - k_2[\mathbf{8}][Cl^-] + k_{-2}[\mathbf{9}] \quad (8d)$$

with

$$[\mathbf{9}] = [\mathbf{2}_{tot}]_0 - [\mathbf{2}_{tot}] - [\mathbf{8}]$$

$$[\mathbf{4}] = [\mathbf{3}]_0 - [\mathbf{3}]$$

$$[\mathbf{2\text{-}D}] = 0.5\,K_{a1}[(1 + 4[\mathbf{2}_{tot}]/K_{a1})^{1/2} - 1]$$

$$[\mathbf{2}] = [\mathbf{2}_{tot}] - [\mathbf{2\text{-}D}]$$

where the index 0 indicates the appropriate value at time = 0.

Simultaneous fit of the theoretical curves for [**3**] to the experimental concentrations from two experiments (fitting of the curve for [**4**] would be redundant, since [**3**] + [**4**] = const.) are shown in Figure 4. The optimized rate constants (Eq. 1) are listed in Table 2 together with those for the reaction (2).

3.3. Kinetics of the reaction between **2** and deoxyguanosine

The reaction between **2** and deoxyguanosine was followed in aqueous 0.1 M $NaClO_4$ at constant pH (4.5±0.1). At this pH, the acid-base equilibria $K_{a1}$, $K_{a2}$ and $K_{a3}$ of Scheme 1 (substitute dGuo for **L** in Scheme 1; beware that the $pK_a$ values are given for $D_2O$) can be neglected, and we are left with the two consecutive reactions along $k_{L1}$ and $k_{BIS}$. The concentration curves for dGuo, *cis*-$[Pt(NH_3)_2(dGuo)(H_2O)]^{2+}$

(**5**), and *cis*-$[Pt(NH_3)_2(dGuo)_2]^{2+}$, determined by reverse-phase HPLC, are shown in Figure 5, and the optimized rate constants are given in Table 1.

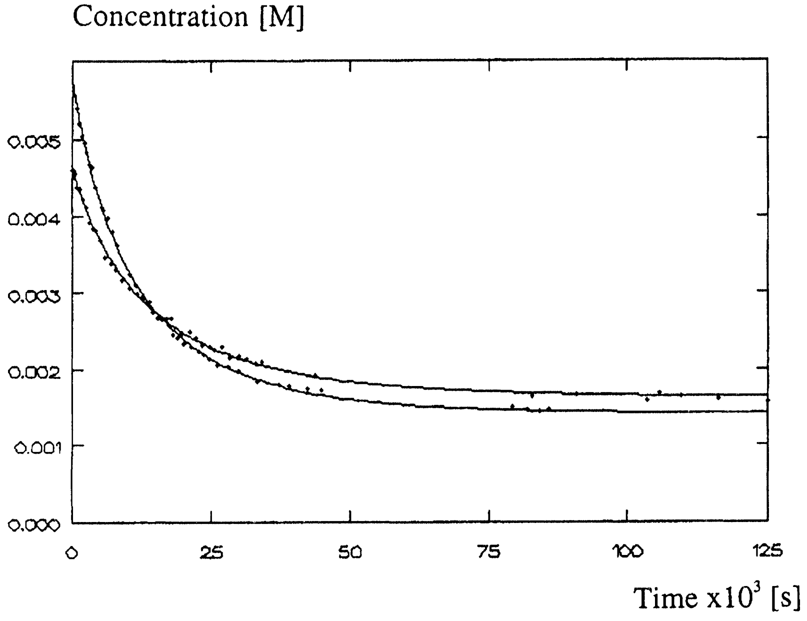

**Figure 4.** Experimental (+) and calculated (full line) concentration curves for [**3**] for two runs in which approx. stoichiometric amounts of **3** and NaCl were mixed in 0.1 M $NaClO_4$ at 20 °C. The rate constants $k_{Cl}$ and $k_{H2O}$ were iterated in order to fit simultaneously both experimental curves.

**Table 2.** Hydrolysis and chloride anation rate constants for the reversible reactions

$$cis\text{-}[Pt(NH_3)_2(H_2O)L]^{(2)+} + Cl^- \underset{k_{H2O}}{\overset{k_{Cl}}{\rightleftharpoons}} cis\text{-}[PtCl(NH_3)_2L]^{(+)} + H_2O \text{ (L = dGuo; Me-5'-dGMP}^-\text{)}$$

Standard deviations are given in parentheses.

| L | dGuo | Me-5'-dGMP⁻ |
|---|---|---|
| $k_{H2O}$ [$s^{-1}$] | $1.3(1)x10^{-5}$ | $0.9(1)x10^{-5}$ |
| $k_{Cl}$ [$M^{-1}s^{-1}$] | $7.5(4)x10^{-2}$ | $1.0(1)x10^{-2}$ |

3.4. Kinetics of the reversible hydrolysis of *cis*-$[PtCl(NH_3)_2(dGuo)]^+$ (**6**)

The monochloro-monodeoxyguanosine complex **6** was isolated from a chloride-rich solution of **5** (see Section 2) using cation-exchange HPLC, and the establishment of the equilibrium (2) in 0.1 M $NaClO_4$ at pH = 4.5±0.1 was followed using analytical cation-exchange HPLC runs. The deprotonation of the aqua ligand of **5** to *cis*-$[Pt(OH)(NH_3)_2(dGuo)]^+$ (**5-H**) could be quantified using the $pK_a$ value determined previously (5.43±0.15 in $D_2O$ [35]; 4.91±0.15 in $H_2O$ [32]):

$$[5] = [5_{tot}]\,[H^+]/(K_a + [H^+]) \qquad (9)$$

with

$$[5_{tot}] = [5] + [5\text{-}H]$$

Eq. (9) yields, with $[H^+] = 10^{-4.5}$ and $K_a = 10^{-4.91}$, 72 % of **5** and 28 % of **5-D**. These percentages have been taken into account in the simulation of the concentration curves for $5_{tot}$ and **6** (Figure 6). It was assumed that the hydroxo form **5-H** is inert towards substitution. Optimization of the rate constants yielded, after optimization, the values listed in Table 2.

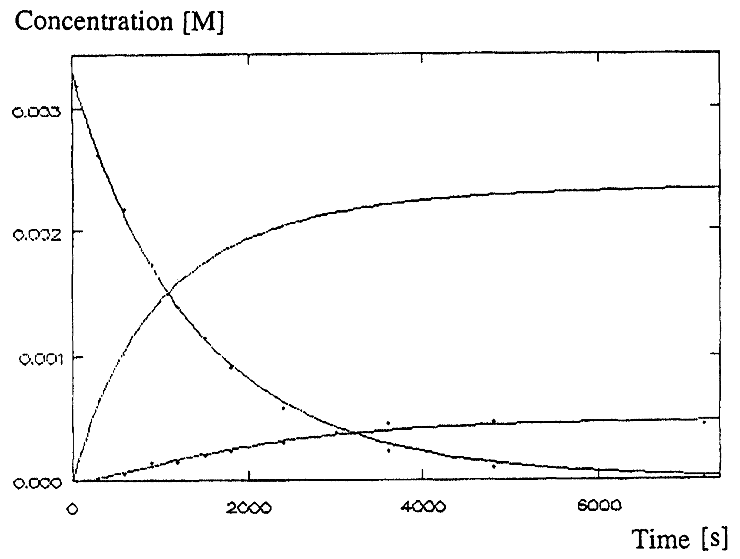

**Figure 5.** Experimental (+) and calculated (full line) concentration curves for deoxyguanosine (descending curve), *cis*-$[Pt(NH_3)_2(dGuo)(H_2O)]^{2+}$ (ascending curve, major final product), and *cis*-$[Pt(NH_3)_2(dGuo)_2]^{2+}$ (ascending curve, minor final product), from a reaction between deoxyguanosine and 2 equivs. of **2** in 0.1 M $NaClO_4$ at 20 °C.

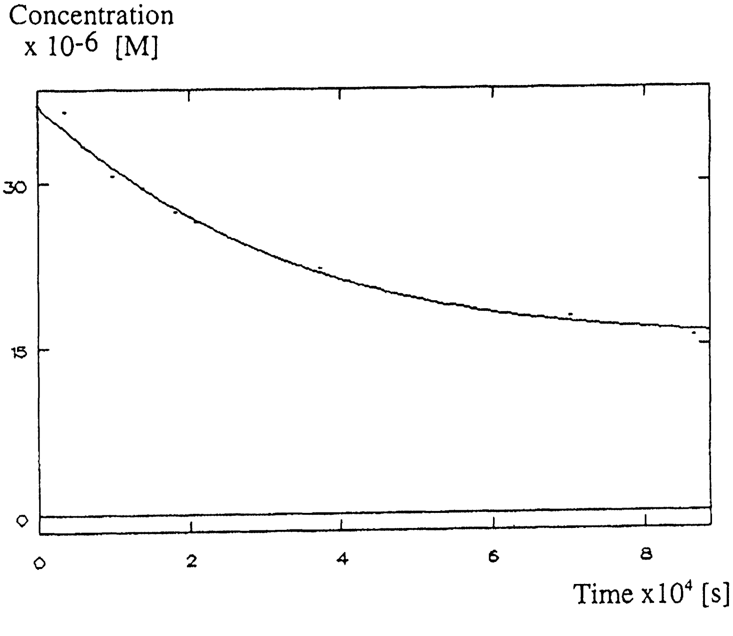

**Figure 6.** Typical experimental (-) and calculated (full line) concentration curves for **6** left to equilibrate in 0.1 M $NaClO_4$ at 20 °C.

### 4. Discussion

4.1. Effect of the attachment of a 5'-methylphosphoester group on the reactivity of deoxyguanosine and its platinum complexes

In the first part of this work (sections 3.1 and 3.3), we evaluated the rate constants for the two consecutive reactions of Me-5'-dGMP$^-$ with *cis*-$[Pt(NH_3)_2(H_2O)_2]^{2+}$ (**2**), and for the analogous reactions of deoxyguanosine. The rate constants (Table 1) show that the substitution of the first aqua ligand of **2** ($k_{L1}$; see Scheme 1) is accelerated by a factor of ~10 by the phosphodiester group. The substitution of the second aqua ligand of **2** ($k_{BIS}$) is accelerated only by a factor of ~2. Two effects are probably responsible for the observed acceleration of the reactions with Me-5'-dGMP$^-$: i) the negative charge of Me-5'-dGMP$^-$, which is expected to favor strongly the interaction with the dicationic species *cis*-$[Pt(NH_3)_2(H_2O)_2]^{2+}$ and somewhat less efficiently that with the monocationic complex *cis*-$[Pt(NH_3)_2(H_2O)(Me\text{-}5'\text{-}dGMP)]^+$; ii) hydrogen bonding between the aqua and ammine ligands of platinum and the 5'-phosphodiester group [36]. Since both effects are expected to be stronger for the first reaction ($k_{L1}$), the smaller rate enhancement observed for $k_{BIS}$, as compared to $k_{L1}$, does not allow to conclude which effect is more important.

For the reaction system involving Me-5'-dGMP$^-$, we have also determined the rate constant $k_{L2}$ characterizing the reaction with the deprotonated form of **2**, *cis*-$[Pt(OD)(ND_3)_2(D_2O)]^+$ (**2-D**) (Scheme 1; Table 1). The ratio $k_{L1}/k_{L2}$ of ~100 indicates that the affinity of **2** for Me-5'-dGMP$^-$ is decreased by two orders of magnitude upon deprotonation of **2**. For comparison, the reaction between **2** and the nucleoside 1-methylinosine is slowed down only by a factor of 10 upon deprotonation [37]. This difference can be plausibly attributed to the negative charge of Me-5'-dGMP$^-$, which should "feel" the decrease of positive charge from **2** to **2-D**, whereas there is no net charge-charge interaction between the platinum complexes and 1-methylinosine. Interpolating between Me-5'-dGMP$^-$ (charge = -1) and 1-methylinosine (charge = 0) to double-stranded DNA, where the average nucleotide charge is approx. -0.25 e [38,39] we would expect that deprotonation of **2** should reduce the reactivity towards DNA nucleobases by a factor between 10 and 100. In contrast to this prediction (as well as to chemical intuition) is the observation reported by Johnson *et al.* that deprotonation of *cis*-$[Pt(NH_3)_2(H_2O)_2]^{2+}$ does not alter its reactivity towards calf thymus DNA [40]. A detailed re-examination of the reactivities of **2** and **2-D** towards duplex DNA would be needed to clarify this point.

In the second part (sections 3.2 and 3.4), we investigated the establishment of the hydrolysis-anation equilibrium for the reactions (1) and (2), respectively. The rate constants shown in Table 2 indicate that whereas the chloride anation of *cis*-$[Pt(NH_3)_2(H_2O)(Me\text{-}5'\text{-}dGMP)]^+$ (**3**) in 0.1 M $NaClO_4$ is ~7 times slower than that of *cis*-$[Pt(NH_3)_2(H_2O)(dGuo)]^{2+}$ (**5**), the reverse hydrolysis reactions proceed with very similar rates. This result seems to invalidate the hypothesis of Kozelka and Barre, according to which the 5'-phosphodiester group adjacent to a guanine coordinating a *cis*-$PtCl(NH_3)_2^+$ residue would act as a nucleophilic catalyst and accelerate in a sequence-dependent manner the hydrolysis of the monoadduct [27].

4.2. Methodological considerations

In this work, two very similar reaction systems were analyzed using two quite different approaches. The system involving deoxyguanosine was investigated classically [31,37] in a reaction vessel from which samples were withdrawn and analyzed by means of HPLC. This setting allows the pH to be measured and adjusted continuously, which considerably simplifies the data analysis. Such a practice becomes problematic, however, when the experiments have to be carried out in neutral solution, where pH adjustments are extremely delicate.

NMR offers the opportunity of performing *in situ* qualitative and quantitative analysis of the products consumed and formed during a reaction. However, no pH adjustments during the reaction are possible with current spectrometers. Workers using NMR for kinetic measurements therefore usually either choose acidic or alkaline reaction conditions involving only small pH changes, or recur to buffers. Buffer systems, however, inevitably include potential ligands, and data recorded for reactions involving metal complexes in buffered solutions are therefore frequently ambiguous. Specifically, the phosphate buffer, typically used to maintain neutral or quasi-neutral pH, has been shown to compete effectively with other nucleophiles for platinum(II) binding [41]. We have therefore followed, in the part involving Me-5'-dGMP$^-$, an alternative approach, in which the pH is not *regulated*, but left to evolve, and the pH changes are *taken explicitly into account* in the kinetic simulation. Two options of this demarche are presented: the theoretical approach, where the concentration of $H^+$ (actually: $D^+$) is calculated from the known dissociation constants, and the experimental approach, employing an internal pD indicator. The application of the theoretical approach was particularly straightforward for the reaction between Me-5'-dGMP$^-$ and **2** in acidic solution (Section 3.1.1.), where only two acid-base equilibria were to be taken into account, and the proton concentration could thus be derived analytically. The same reaction in neutral medium, on the other hand, involved three protolytic equilibria (Scheme 1), and the calculation of $[D^+]$ would have had to be carried out numerically. The introduction of an iterative subroutine within each cycle of the routine integrating the differential equations would have considerably slowed down the integration-optimization procedure and would have possibly jeopardized the convergence. Therefore, we recurred to the experimental approach. The experimental $[D^+]$-time curve was introduced into the integration routine by means of a fit function (Figure 2).

In summary, replacing pH control by mathematical accounting for pH changes in the evaluation of the rate constants has allowed us to study in a convenient way a relatively complicated system of reactions.

This approach requires only a moderately increased computational effort, which should be manageable by any chemist familiar with basic algebra.

**Acknowledgment**

We are indebted to Mrs. Bettina Beck and Professor Erich Dubler for the thermoanalysis of $NH_4Me$-5'-dGMP, Johnson-Matthey for a generous loan of platinum complexes, and the IDRIS center of the Centre National de la Recherche Scientifique for computer time. Financial support from the COST scientific exchange program D8/0004/97 and from the Ligue Nationale Française contre le Cancer (grant accorded to F. L.) is gratefully acknowledged.

**References**

[1] M. J. Bloemink and J. Reedijk, *Met. Ions Biol. Syst., 32* (1996) 641-685.
[2] J. Zlatanova, J. Yaneva and S. H. Leuba, *FASEB J., 12* (1998) 791-799.
[3] C. A. Lepre and S. J. Lippard, *Nucleic Acids Mol. Biol., 4* (1990) 9-38.
[4] L. J. N. Bradley, K. J. Yarema, S. J. Lippard and J. M. Essigman, *Biochemistry, 32* (1993) 982-988.
[5] K. J. Yarema, S. J. Lippard and J. M. Essigman, *Nucleic Acids Res., 23* (1995) 4066-4072.
[6] E. Reed, R. F. Ozols, R. Tarone, S. T. Yuspa and M. C. Poirier, *Proc. Natl. Acad. Sci. USA, 84* (1987) 5024-5028.
[7] P. Mailliet, E. Segal-Bendirdjian, J. Kozelka, M. Barreau, B. Baudoin, M.-C. Bissery, S. Gontier, A. Laoui, F. Lavelle, J.-B. Le Pecq and J.-C. Chottard, *Anti-Cancer Drug Design, 10* (1995) 51-73.
[8] P. Augé and J. Kozelka, *Transition Met. Chem., 22* (1997) 91-96.
[9] B. Lambert, J.-L. Jestin, P. Bréhin, C. Oleykowski, A. Yeung, P. Mailliet, C. Prétot, J.-B. Le Pecq, A. Jacquemin-Sablon and J.-C. Chottard, *J. Biol. Chem., 270* (1995) 21251-21257.
[10] M. Coluccia, A. Nassi, F. Loseto, A. Boccarelli, M. A. Marriggiò, D. Giordano, F. P. Intini, P. A. Caputo and G. Natile, *J. Med. Chem., 36* (1993) 510-512.
[11] M. Coluccia, M. A. Marriggio, A. Boccarelli, F. Loseto, N. Cardellicchio, P. Caputo, F. P. Intini, C. Pacifico and G. Natile (1996) in Pinedo H. M. and J. H. Schornagel (ed.), *Platinum and Other Metal Coordination Compounds in Cancer Chemotherapy,* Plenum Press, New York, London, pp. 27-35.
[12] N. Farrell, L. R. Kelland, J. D. Roberts and M. van Beusichem, *Cancer Res., 52* (1992) 5065-5072.
[13] M. Van Beusichem and N. Farrell, *Inorg. Chem., 31* (1992) 634-639.
[14] V. Brabec, O. Vrána, O. Nováková, V. Kleinwachter, F. P. Intini, M. Coluccia and G. Natile, *Nucleic Acids Res., 24* (1996) 336-341.
[15] R. Žaludová, A. Žákovská, J. Kašpárková, Z. Balcarová, O. Vrána, M. Coluccia, G. Natile and V. Brabec, *Mol. Pharmacol., 52* (1997) 354-361.
[16] A. Žákovská, O. Nováková, Z. Balcarová, U. Bierbach, N. Farrell and V. Brabec, *Eur. J. Biochem., 254* (1998) 547-557.
[17] P. Horáček and J. Drobník, *Biochim. Biophys. Acta, 254* (1971) 341-347.
[18] D. L. Bodenner, P. C. Dedon, P. C. Keng and R. F. Borch, *Cancer Res., 46* (1986) 2745-2750.
[19] D. P. Bancroft, C. A. Lepre and S. J. Lippard, *J. Am. Chem. Soc., 112* (1990) 6860-6871.
[20] R. J. Knox, F. Friedlos, D. A. Lydall and J. J. Roberts, *Cancer Res., 46* (1986) 1972-1976.
[21] J.-M. Malinge and M. Leng, *Nucleic Acids Res., 16* (1988) 7663-7672.
[22] F. Reeder, F. Gonnet, J. Kozelka and J. C. Chottard, *Chem. Eur. J., 2* (1996) 1068-1076.
[23] S. J. Berners-Price, K. J. Barnham, U. Frey and P. J. Sadler, *Chem. Eur. J., 2* (1996) 1283-1291.
[24] F. Legendre, J. Kozelka and J.-C. Chottard, *Inorg. Chem., 37* (1998) 3964-3967.
[25] J. Kozelka, F. Legendre, F. Reeder and J.-C. Chottard, *Coord. Chem. Rev.,* (1999) in press.
[26] D. Payet, F. Gaucheron, M. Šíp and M. Leng, *Nucleic Acids Res., 25* (1993) 5846-5851.
[27] J. Kozelka and G. Barre, *Chem. Eur. J., 3* (1997) 1405-1409.
[28] H. J. S. King, *J. Chem. Soc.,* (1938) 1338-1346.
[29] B. Lippert, C. J. L. Lock, B. Rosenberg and M. Zvagulis, *Inorg. Chem., 16* (1977) 1525-1529.
[30] S. K. Miller, D. G. VanDerveer and L. G. Marzilli, *J. Am. Chem. Soc., 107* (1985) 1048-1055.
[31] F. Gonnet, J. Kozelka and J.-C. Chottard, *Angew. Chem. Int. Ed. Engl., 31* (1992) 1483-1485.
[32] R. B. Martin, *Science, 139* (1963) 1198-1203.
[33] S. J. Berners-Price, T. A. Frenkiel, U. Frey, J. D. Ranford and P. J. Sadler, *J. Chem. Soc. Chem. Commun.,* (1992) 789-791.
[34] W. H. Press, B. P. Flannery, S. A. Teukolsky and W. T. Vetterling (1989) *Numerical Recipes,* Cambridge University Press, Cambridge.
[35] D. Lemaire, M.-H. Fouchet and J. Kozelka, *J. Inorg. Biochem., 53* (1994) 261-271.
[36] M. Green, M. Garner and D. M. Orton, *Transition Met. Chem., 17* (1992) 164-176.

[37] J. Arpalahti, *Inorg. Chem., 29* (1990) 4598-4602.
[38] C. F. Anderson, M. T. Record, Jr. and P. A. Hart, *Biophys. Chem., 7* (1978) 301-316.
[39] G. S. Manning, *Quart. Rev. Biophys., 11* (1978) 179-246.
[40] N. P. Johnson, J. D. Hoeschele and R. O. Rahn, *Chem.-Biol. Interact., 30* (1980) 151-159.
[41] T. G. Appleton, R. D. Berry, C. A. Davis, J. R. Hall and H. A. Kimlin, *Inorg. Chem., 23* (1984) 3514-3521.

**Received: November 9, 1998 - Accepted: November 27, 1998 - Received in revised camera-ready format: December 16, 1998**

